# Thirty-sixth Annual Commencement of the Atlanta Medical College

**Published:** 1894-03

**Authors:** 


### Editorial Notes.

Dr. Joseph Price, of Philadelphia, has resigned as physician-in-charge of the Preston Retreat, and Dr. Richard C. Norris, associate editor of the *Annals of Gynecology and Pediatry*, has been elected his successor.

The Medical Society of the State of New York.—The following officers were elected for the ensuing year: President, George Henry Fox, of New York; Vice-President, Dr. Frank S. Low, of Pulaski; Secretary, Dr. Frederic C. Curtis, of Albany; Treasurer, Dr. Charles H. Porter, of Albany.

*To Editors Southern Medical Record:*

It has been insinuated to me during the past year by two of our most prominent opticians that I might get a rebate of twenty-five per cent. on prescriptions for glasses sent them. They explain that the thing is done by some of our scientific oculists and regard it (very correctly from their standpoint) as a purely business proposition. I trust they have misrepresented the facts, as it would really be a humiliation to know that a scientific oculist or physician would so far forget the ethical part of his profession as to enter into a *combination* of any kind, for mutual benefit. The same thing has been insinuated, or stated, with reference to some of our druggists. May we not hope, Mr. Editor, that it is all a mistake?

F. H. Sims.

The visit of several of the editors of our Northern and Eastern contemporaries was a source of much pleasure to us, and it is with genuine regret that their visit was of such short duration.

It is seldom that medical journalists tear themselves away from the many vexations which surround them to take a week off in quest of the pleasure and relaxation which was experienced by our visitors.

The cordiality with which they were received at the several points of destination attested their brilliancy, popularity and good fellowship.

We shall ever retain many pleasant memories of our distinguished friends and it is our sincere wish that their visit may be often repeated.

Expressions of amazement and delight were heard of our glorious climate and the many advantages of our Southern country as a resort for the invalid from the chilling blasts of the Northern countries.

The intermingling and association with these eminent gentlemen, even for so short a period, is of great value to both sections, and we expect to see great good come from their opinions as expressed through the medium of their publications. The party was met here by a committee from the Atlanta Society of Medicine and were escorted to the Commercial Club rooms where a reception was given them. Here they met local members of the profession and many of the representative business men of the city.

The party consisted of Dr. W. C. Wile, *New England Monthly*, Danbury Conn.; Dr. Howard Van Rensselaer, *Medical Annals*, Albany, N. Y.; Dr. W. Blair Stewart, *Medical Bulletin*, Philadelphia; Dr. Ferdinand King, New York *Polyclinic*, New York; Hon. Clark Bell, *Medico-Legal Journal*, New York; Mr. Stewart Griffing, New Haven, Conn; Dr. L. J. Picot, Littleton, N. C.; Dr. G. Stewart, Physician-in-chief, Ward's Island, New York, and Rev. Dr. McNeal, of Bridgeport, Conn., who represented the *Daily Standard* of that city.

---

### THIRTY-SIXTH ANNUAL COMMENCEMENT OF THE ATLANTA MEDICAL COLLEGE.

Sixty-nine new doctors were turned out from the Atlanta Medical College on Tuesday night, March 6th.

The graduating exercises, which were very entertaining, were held in the Grand.

The opening prayer was made by Rev. Dr. C. P. Williamson.

### THE PROCTOR'S REPORT.

Dr. W. S. Kendrick, the Proctor of the College, made the following report for the Faculty to the Board of Trustees, which was very gratifying to the friends of the institution :

"Mr. President and Gentlemen," said he, "in behalf of the faculty of the Atlanta Medical College, I submit the following report: The session closing with these exercises has been one of unusual prosperity and success. The matriculation book shows an enrollment of three hundred students in the various departments."

Following the Proctor's report, Colonel N. J. Hammond, President of the Board of Trustees, conferred the degrees upon the young doctors.

### A LIST OF THE GRADUATES.

The following is a full list of the graduating class:

J. E. Anderson, J. H. Asher, W. F. Ashmore, D. W. Baggs, J. B. Bagley, J. P. Bell, E. P. Bomer, E. F. Bourquin, E. W. Boyd, C. P. Brannen, T. B. Bush, H. C. Call, M. G. Campbell, J. H. Carter, W. M. Carter, J. B. Carmical, J. E. Chalk, J. L. Cloud, E. L. Cosby, J. S. Cousins, A. H. Culpepper, J. W. Daniels, J. B. Dillard, J. W. Dorough, D. H. Durham, O. H. Fleming, H. F. Frederick, W. V. Garrett, O. N. Hardon, E. F. Harris, J. E. T. L. Hayes, H. H. Henderson, W. P. Henry, W. T. Hinton, T. S. Holleyman, T. C. Jeffords, W. C. Kennedy, L. V. Lee, J. A. Lester, H. L. Miller, H. G. Minter, J. M. Moore, Daniel Moore, J. R. Palmer, C. E. Pickett, W. A. J. Pollock, Jr., B. F. Reeves, L. L. Richardson, J. C. Riley, W. A. Rivers, J. L. Roberts, W. D. Rogers, W. R. Russell, J. L. Sonborn, W. C. Stovall, T. W. Tate, W. C. Thomas, W. A. Tripp, O. H. Weaver, Anthony White, L. A. Williams, K. S. Williams. J. E. Woods, L. O. Wooten, F. A. Wynn, D. H. Yates.

Pharmacy—W. S. Schrayer, Nathan Schrayer, R. C. Hood, O. B. Hartzog, A. R. Moody.

The annual address was delivered by Dr. F. M. Ridley of LaGrange. The doctor is one of the most forcible and eloquent speakers in the State, as well as one of the leading physicians, and his address Tuesday night was a beautiful production.

The valedictory address, which was an able one, was delivered by Dr. J. L. Roberts, and he was frequently interrupted by applause.

### THE PRIZES AWARDED.

Mr. George Westmoreland, in a very graceful manner, delivered the prizes to the successful contestants.

Dr. Moses Gatlin Campbell won the first honor, Dr. William Pickens Henry won the second and Dr. Olin Heard Weaver won third.

Favorable mention was made of Dr. J. M. Moore, Dr. C. E. Pickett, Dr. J. W. Dorough, Dr. H. F. Frederick and Dr. J. H. Asher.

